# Room Temperature UV Photodetector Based on Aero-Titania

**DOI:** 10.3390/ijms262211035

**Published:** 2025-11-14

**Authors:** Mircea Nicolaescu, Tudor Braniste, Corina Orha, Mina-Ionela Morariu, Sebastian Lehmann, Kornelius Nielsch, Ion M. Tiginyanu, Raluca Faur, Victor Zalamai, Carmen Lazau, Cornelia Bandas

**Affiliations:** 1 Condensed Matter Department, National Institute for Research and Development in Electrochemistry and Condensed Matter, 300224 Timisoara, Romania; nicolaescu.mircea13@yahoo.com (M.N.); orha.corina@gmail.com (C.O.); mina.popescu@student.upt.ro (M.-I.M.); carmen.lazau@gmail.com (C.L.); 2 National Center for Materials Study and Testing, Technical University of Moldova, 2004 Chisinau, Moldova; tiginyanu@gmail.com (I.M.T.); victor.zalamai@cnstm.utm.md (V.Z.); 3 Institute for Metallic Materials (IMW), Leibniz Institute of Solid State and Materials Research (IFW Dresden), Helmholtzstrasse 20, 01069 Dresden, Germany; s.lehmann@ifw-dresden.de (S.L.); k.nielsch@ifw-dresden.de (K.N.); 4 Academy of Sciences of Moldova, 2001 Chisinau, Moldova; 5 National Research & Development Institute for Welding and Material Testing-ISIM Timisoara, 300222 Timisoara, Romania; rfaur@isim.ro

**Keywords:** sensor, aero-titania, zinc oxide template, ultraviolet detection

## Abstract

This research demonstrates, for the first time, the integration of aero-titania material in sensor devices. An innovative approach for the practical application of aero-titania (aero-TiO_2_) materials in photodetectors and the characterization under ultraviolet irradiation was assessed. The fabrication of aero-materials was carried out through the atomic layer deposition (ALD) of titanium dioxide ultrathin layers on a sacrificial network consisting of zinc oxide micro-tetrapods. This process was followed by a selective etching of the sacrificial ZnO template and formation of aero-titania hollow micro-tetrapods. The obtained material has been characterized using UV-Vis spectroscopy, scanning electron microscopy (SEM), and X-ray diffraction (XRD) analysis. The development of photodetectors was achieved through the sequential spin-coating deposition of aero-TiO_2_ onto an interdigitated ceramic electrode. The obtained results show that, for high-intensity ultraviolet, the maximum sensitivity was reached for the two-deposited-layer aero-TiO_2_ sensor at about 23, since for the low-intensity UV the highest sensitivity was recorded for the one-deposited-layer aero-TiO_2_ sensor at about 12. In terms of the responsivity, the highest response was obtained for the one-deposited-layer aero-TiO_2_ sensor under low-intensity illumination, reaching about 1.23 × 10^−4^ A W^−1^ cm^−2^. Thus, the aero-TiO_2_ structure demonstrates the practical viability and application potential of this emerging class of materials in advanced sensing technologies.

## 1. Introduction

The development of ultraviolet (UV) radiation detectors using nanostructured materials is of great interest, considering the various applications including environmental monitoring, healthcare, and security. UV nano-sensors are characterized by several key features that make them highly effective for detecting ultraviolet radiation. The nanoscale sensors exhibit an exceptional sensitivity, allowing for the detection of very low levels of UV light with high precision and short response times, enabling the real-time monitoring of radiation levels. The small size facilitates integration into compact devices, such as wearable technology or embedded systems. Often, the development of UV nano-sensors focuses on materials like zinc oxide (ZnO) and titanium dioxide (TiO_2_), known for their strong UV absorption and photo-reactivity. These materials can be engineered at the nanoscale to improve their responsiveness to UV light [1].

Due to their amazing properties, aero-materials have attracted increasing interest from researchers and have been applied in various fields. Comparatively to aerogels, these porous material types are produced using different technologies, involving the use of sacrificial templates that are later removed. This process results in extremely porous materials with unique characteristics. After the template is removed, the ultra-light and highly porous aero-materials almost entirely replicate the shape of the original template [2,3]. Numerous approaches have been used to study metal oxide material nanostructures from the perspectives of synthesis, structure, and properties. Agglomerations and airborne durability have been identified as critical issues in the synthesis process that determine the size, shape, and crystal orientations of nanostructures [4]. ZnO is a wide bandgap semiconductor compound widely used in practical applications due to its chemical and optical properties. ZnO nanoparticles (ZnO NPs) are a versatile class of nanomaterials with diverse applications due to their unique properties, such as a high surface area, low toxicity, and direct band gap, making them useful in areas like sunscreens, drug delivery, and antimicrobial applications. Although the material is relatively easy to synthesize in a very large variety of shapes, the applicability of ZnO nano-micro-architectures is limited due to the low chemical stability of the material in basic and acidic environments.

In recent years, ZnO nanostructures such as nanowires [5,6], nanorods [7,8], nanoparticles [9,10], and tetrapods [11,12] have been used as sacrificial templates to obtain porous materials. Of all of these ZnO nanostructures, the tetrapod’s shape was highlighted in recent years for its distinctive 3D structure. This geometry not only provides exceptional stability—thanks to the way force applied to one arm is distributed across the others—but also allows for easy access and manipulation at the nanoscale [13]. Current research exhibits that assembling nanoscale components into complex 3D architectures leads to significantly improved properties compared to 1D structures. In the literature, ZnO tetrapods were obtained using various techniques, including atmosphere-controlled oxidation, thermal evaporation, catalytic oxidation, microwave radiation, and microemulsion [14], attracting considerable attention due to their shape, good optical properties, and low density. Zinc oxide (t-ZnO) nano- and micro-tetrapods have exhibited a promising potential in many applications, including biomedical engineering, optoelectronic and nanoelectronic sensing, energy harvesting and storage, and environmental protection. ZnO tetrapod particles can be assembled to form large, highly porous structures and, due to their long extensions arms, inhibit tight packing and result in a high free volume. These interconnected structures provide mechanical stability, flexibility, low density, and easy access to active surfaces [15].

Among oxide materials, TiO_2_ is a versatile compound known for its brightening and UV-absorbing properties, non-toxicity, and chemical stability. As a photocatalyst, particularly effective in its anatase phase, TiO_2_ is used in environmental purification, sunscreen, self-cleaning surfaces, and energy applications like dye-sensitized solar cells [16]. Its synthesis involves techniques such as sol–gel processing, hydrothermal methods, electrochemistry, and physical vapor deposition. Nanostructured titanium dioxide, offering an enhanced surface area and reactivity, is synthesized through advanced methods like anodic oxidation, and is applied in diverse fields, including enhanced photocatalysis, biomedical applications, and optoelectronics, promising advancements in sustainable and high-tech applications [17].

In our previous works, we demonstrated that aero-TiO_2_ materials can be synthesized using a sacrificial network of ZnO micro-tetrapods as a template. The process involved coating the ZnO network with a thin film of TiO_2_ via atomic layer deposition (ALD), followed by two key steps: thermal treatment at varying temperatures and the selective etching of the ZnO template. The etching process can be performed using two different methods: wet etching in a citric acid solution and vapor-phase etching at a high temperature in a hydride vapor-phase epitaxy (HVPE) system using HCl and hydrogen gases. The resulting aero-TiO_2_ materials exhibited a controlled morphology, composition, and crystal structure, dependent on the annealing temperature and the specific sequence of the technological steps [18]. In this work, we demonstrate the application of aero-TiO_2_ nanomaterial as an efficient highly sensitive ultraviolet detector in air at room temperature. Furthermore, the aero-titania-based nanomaterials used in these sensors possess an enhanced stability and durability under various environmental conditions, ensuring long-term reliability.

## 2. Results and Discussion

### 2.1. Investigation of Morpho-Structural Properties

**Figure 1:**
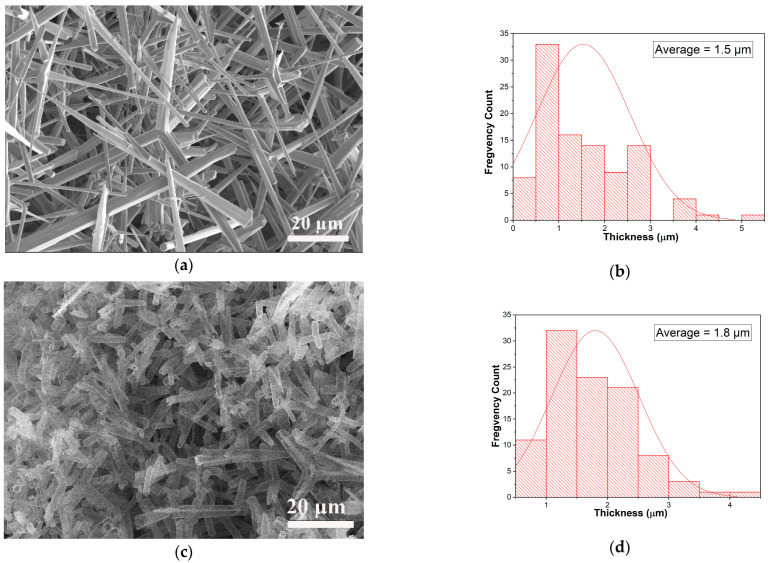
SEM pictures of the initial template made of an interconnected network of ZnO micro-tetrapods (**a**) and the resulting aero-titania after ALD of TiO_2_ layers and removal of ZnO substrate (**c**). Histogram of the average size for ZnO micro-tetrapods (**b**) and aero-titania (**d**).

The detailed morphology of the aero-nanomaterials was revealed using scanning electron microscopy (SEM). Figure 1a shows the SEM image of the interconnected network of ZnO micro-tetrapods used as a sacrificial template for TiO_2_ synthesis, while the obtained aero-TiO_2_ nanomaterials are presented in Figure 1c. Using ImageJ (Version 1.53t) software, the arm diameter distributions of the ZnO template (Figure 1b) and the synthesized aero-titania (Figure 1d) were calculated. The histograms presented in Figure 1b,d show the average size of ZnO and aero-TiO_2_ arms varying in the range of 1–3 µm.

**Figure 2:**
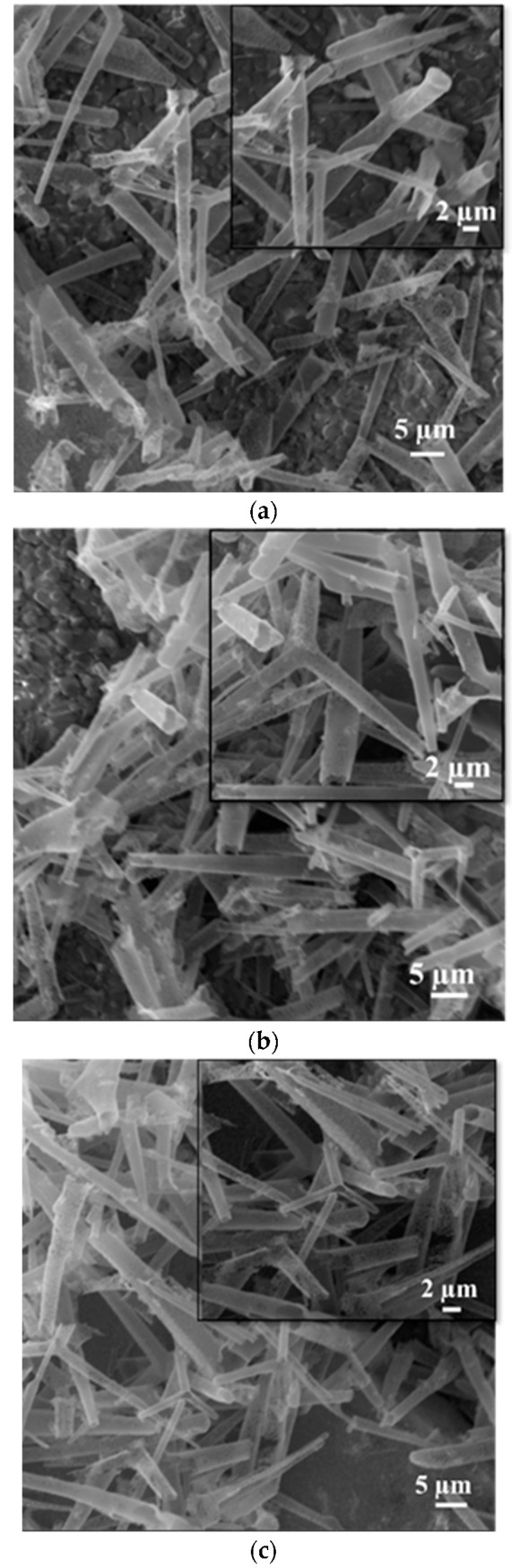
SEM images of the interdigitated electrodes deposited with aero-titania through the spin-coating method: one deposited layer (**a**), two deposited layers (**b**), and three deposited layers (**c**).

The homogenous distribution of the aero-titania on the surface of the interdigitated electrodes after several cycles of spin-coating was assessed using scanning electron microscopy. Figure 2 shows the as-deposited aero-TiO_2_ on the gold interdigitated electrode, in accordance with the number of spin-coating cycles, a visible increase in tetrapod density can be observed.

**Figure 3:**
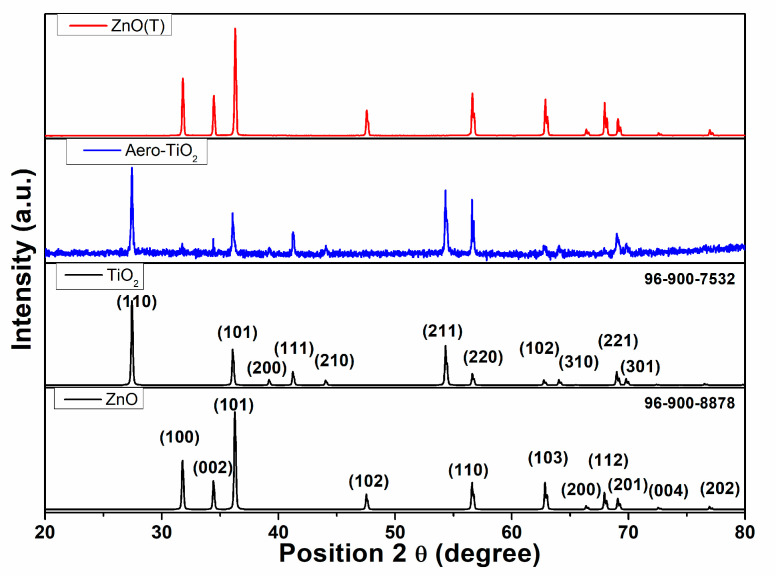
The XRD patterns of the initial ZnO micro-tetrapods and the resulting aero-TiO_2_, confirmed by JCPDS card no. 96-900-8878 and card no. 96-900-7535 materials, respectively.

To determine the crystallinity of the materials, X-ray diffraction (XRD) analysis was performed (Figure 3). Figure 3 presents the XRD spectra for the (ZnO(T)) template and the as-formed aero-composite derived from aero-TiO_2_ following the atomic layer deposition (ALD) of TiO_2_, subsequent acid etching, and thermal treatment. During the etching process, a residual ZnO core remained inside the hollow microtubes of the TiO_2_ layer.

The ZnO(T) was confirmed by JCPDS card no. 96-900-8878, demonstrating a high crystallinity. This is evidenced by the main XRD peaks at 2theta of 31.80°, 34.40°, 36.30°, 47.50°, 56.60°, 62.90°, 66.40°, 67.90°, 69.10°, 72.60°, and 77.0°, which correspond to the (100), (002), (101), (102), (110), (103), (200), (112), (201), (004), and (202) crystal planes, respectively, confirming its hexagonal wurtzite structure [19]. Following the synthesis of the aero-TiO_2_ material, the ZnO crystallized at 2theta of 31.80°, 34.40°, 36.30°, and 69.10°, which correspond to the (100), (002), (101), and (201) crystal planes. This demonstrates a slight preferential etching on the crystallographic planes. The rutile TiO_2_ crystallization in the tetragonal form is confirmed by JCPDS card no. 96-900-7535, with the diffraction peaks at 2theta of 27.4°, 36.1°, 39.2°, 41.2°, 44.0°, 54.3°, 56.6°, 62.7°, 64.0°, 68.9°, and 69.8°, assigned to the (110), (101), (200), (111), (210), (211), (220), (102), (310), (221), and (301) crystal planes, respectively [20]. In a previous scientific report, V. Ciobanu et al. [18] demonstrated the formation of aero-TiO_2_ by depositing ultrathin layers of TiO_2_ onto interconnected networks of ZnO micro-tetrapods using the ALD method. The TiO_2_ deposition at 150 °C was followed by a chemical etching of a sacrificial substrate of ZnO; then, depending on the annealing temperature, the aero-TiO_2_ may be obtained either in anatase or rutile phases. The presence of ZnO peaks in the XRD pattern indicates the incomplete etching of the ZnO substrate during the H_2_ treatment at 800 °C. In our study, the synthesis parameters prevented the formation of the anatase phase, resulting in a complete conversion to the rutile phase under our experimental conditions.

Based on the Debye–Scherrer formula, the crystallite size was calculated for the three most intense diffraction peaks for both compounds. The values and the corresponding $D_{\left(hkl\right)}\;$ indices are presented in Table 1. Furthermore, the average crystallite size was calculated for these selected planes to highlight the need for anisotropy correction when accounting for shape effects in nanostructured tetrapod morphologies. This implies that the calculated crystallite size requires a shape anisotropy correction of ±14.38% for TiO_2_ and ±23.73% for ZnO, respectively. The larger crystallite size of ZnO indicates the incomplete etching of the ZnO substrate during the H_2_ treatment at 800 °C, which results in the formation of a nanocrystalline TiO_2_ shell and some traces of ZnO micro-tetrapods inside of the aero-titania.

Also, Table 1 presents the average crystallite size calculated using the Williamson–Hall (W-H) analysis model, highlighting the effect of micro-strain on the crystallite size. Comparing the Scherrer and Williamson–Hall methods, it can be noted that the inclusion of strain has a significant impact on the calculated average crystallite size. For TiO_2_, the positive micro-strain results in a smaller size compared to Scherrer, while ZnO exhibits a negative micro-strain, leading to a larger W-H size than Scherrer [21].

### 2.2. Optical and Electrical Characteristics

**Figure 4:**
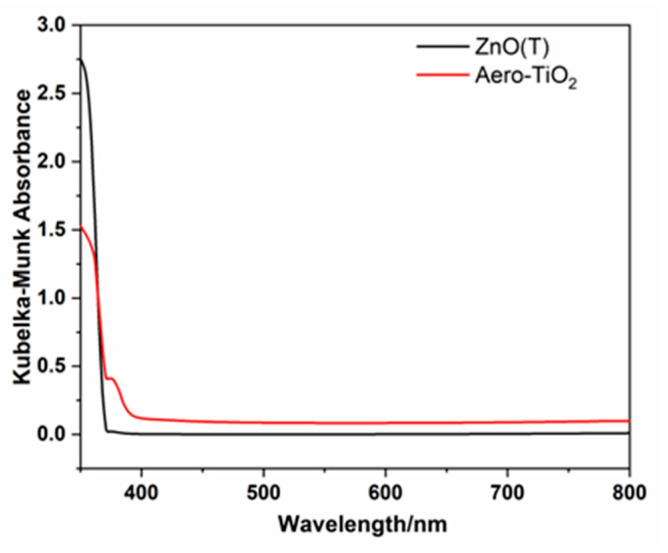
Spectra for Kubelka–Munk absorbance of ZnO(T) and aero-TiO_2_ material.

The optical characteristics of ZnO tetrapods (ZnO(T)) and the aero-TiO_2_ composite were evaluated with UV-vis spectroscopy, and are illustrated in Figure 4. In our case of powder-like samples, the optical diffuse reflectance spectra of our samples measured using an integrating sphere should be converted in a pseudo-absorption function F(R) through the Schuster–Kubelka–Munk approximation: F(R) = (1 − R)/2R, where R is the reflectance of the samples, considering that F(R) corresponds to the optical absorption coefficient α and given the direct bandgap of Rutile-TiO_2_ (confirmed though XRD measurements) in which its E derives from a “hνα versus photon energy hν” plot. Pristine ZnO(T) exhibited a sharp absorption edge around 350 nm, showing a blue shift compared to other studies. Instead, some researchers demonstrated in their comprehensive study that the absorption of ZnO tetrapods is typically around 380 nm [22]. This decrease may be due to the high crystallinity of the tetrapods, compared with bulk ZnO materials around 3.36 eV. After the deposition of TiO_2_ and the ZnO reduction through a thermal treatment, a slight red shift in the absorption edge was observed around 370–390 nm, characteristic for the lower band gap of rutile TiO_2_ and interfacial interactions. This slight extension of the absorption in the near-visible UV region suggests the formation of a core–shell aero-TiO_2_ structure that generated a modification of the optical response [23,24].

**Figure 5:**
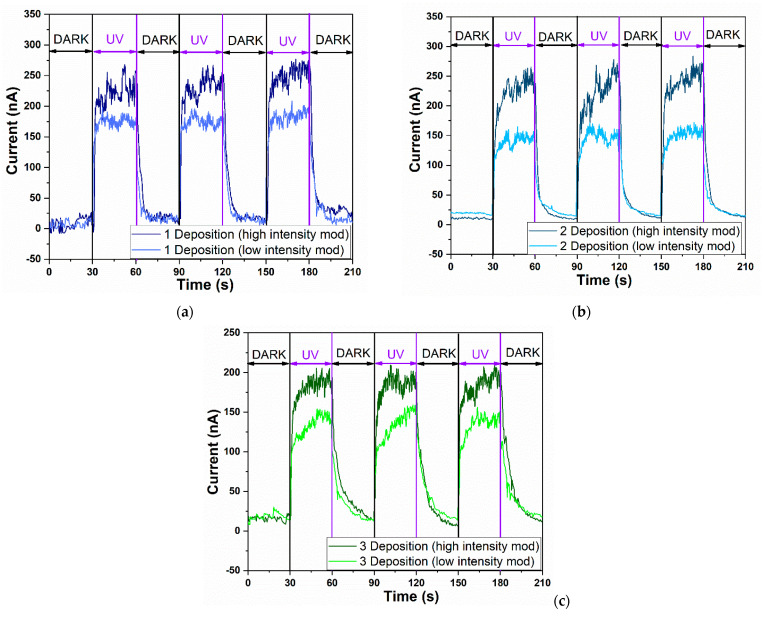
The UV time-dependent current response of aero-titania depending on experimental program at different intensity illumination mode: (**a**) A1-aero-TiO_2_; (**b**) A2-aero-TiO_2_; (**c**) A3-aero-TiO_2_.

In order to compare the electrical behavior of the aero-TiO_2_ sensor under DARK and UV illumination conditions depending on the number of deposition layers (one, two, and three layers), the *I-T* analysis was performed. Figure 5 presents the photodetection properties under different deposition conditions. The aero-TiO_2_ devices were tested under alternating UV and DARK conditions, with 20 s switching intervals at a bias voltage of 10 V. Furthermore, to demonstrate the effect of UV intensity on the materials, the devices were tested under two modes, high-intensity mode (HIM) at 3500 µW cm^−2^ and low-intensity mode (LIM) at 2000 µW cm^−2^, in a normal atmospheric condition.

The sensitivity and responsivity were calculated from the ON/OFF ratio at a 10 V bias using Equations (1) and (2) [25,26].

(1) $$S=I_{UV}/I_{DARK}$$

(2) $$R=\left(I_{UV}-I_{DARK}\right)/\left(P_{opt}A\right)$$

where *I_UV_* and *I_DARK_* represent the current in the presence of photoirradiation and the absence of photo irradiation, respectively, *A* is the effective device area (0.7 cm^2^), and *P* is the optical power density of the UV source (3500 µW cm^−2^ or 2000 µW cm^−2^).

The sensitivity of the sensor, also known as the ON/OFF ratio, exhibits different behavior depending not only on the number of deposition layers, but also on the UV intensity mode. Under high-intensity UV, the highest sensitivity was observed for the A2-aero-TiO_2_ device, reaching a value of 23.6. Under low-intensity UV, the maximum sensitivity was recorded for the A1-aero-TiO_2_ device, with a value of 12.5. Compared to the study by J. Gröttrup et al., which investigated the UV detection properties of hybrid ZnO/ZnAl_2_O_4_ tetrapods in 3D networks, our devices demonstrated an almost fivefold increase in sensitivity under a low-intensity conditions [27]. These findings highlight the need for optimization in achieving high-sensitivity devices. Furthermore, our results emphasize that the deposition process should depend on the intensity of UV illumination. Similar optimization requirements were also reported by Postica V. et al., which also demonstrated the influence of morphology and surface modification on the UV detection performance of ZnO, showing a direct correlation between the structural morphology and detection sensitivity [28].

Furthermore, when examining the retention rate of the photocurrent depending on the UV intensity, the A1-aero-TiO_2_ device exhibits a retention rate of 73.53%, showing good evidence of photocurrent transformation efficiency. We believe this is due to the efficient heterojunction transport at the aero-TiO_2_ interface, as evidenced by the lower turn-on voltage observed in this device. This reduced turn-on voltage, associated with a higher sensitivity, has also been showcased in other research studies [28,29]. Moreover, the responsivity under UV light was calculated based on Equation (2) at different lamp power intensities. Since responsivity is directly influenced by the optical power, the highest value was obtained for the A1-aero-TiO_2_ device under low-intensity illumination, reaching 1.23 × 10^−4^ A W^−1^ cm^−2^. This indicates a better efficiency in converting optical signals into electrical signals, which is consistent with the observed sensitivity value.

By comparing the calculated responsivity values, we observe that, for the A1-aero-TiO_2_ and A2-aero-TiO_2_ devices under both UV illumination modes, the values remain nearly constant, with only a slight variation for A1-aero-TiO_2_ under low-intensity illumination. A notable decrease in responsivity was observed for the A3-aero-TiO_2_ device, which provides direct evidence that increasing the thickness of the aero-TiO_2_ layer and the number of spin-coating cycles reduces the active surface area, thereby decreasing the efficiency of the photo-electric signal conversion. Based on the literature data, the response time is defined as the time required for the sensor to reach 90% of the maximum photocurrent, while the time required for the photocurrent to decrease to 10% of its maximum value is referred to as the recovery time [30]. For the investigated devices, the response time was near the same range for all three types of sensors, at around 2.5 s. A more noticeable variation in the device was observed in terms of the recovery time, with the A2-aero-TiO_2_ device exhibiting the fastest recovery in both intensity modes, at 4.4 s for HIM and 3.2 s for LIM. The improvement in recovery speed resulted from the increase in active surface area, which favored light trapping and was influenced by the number of layers and furthermore enhanced junction interface dynamics. To explain the methods of improving sensors and, implicitly, increasing sensitivity, D. R. Miller and his team identified three main factors that enhance detection performance: (1) electrical effects at the interface, (2) chemical effects at the surface, and (3) morphological effects [1].

A slight modification of the DARK current was observed, increasing from ~9 nA to ~22 nA. This change can be attributed to the variation in the number of deposition layers, which affects the residence as a result of increasing the film thickness.

The combined electrical effects at the interface in the highly porous nanocrystalline aero-TiO_2_ lead to the efficient separation of charge carriers and the formation of potential barriers that enhance UV sensitivity. At the same time, the 3D morphology of the tetrapod increased the specific active surface area, facilitating an extended interaction with UV radiation [31,32,33]. However, in the Supplementary Materials, Figure S1 presents the *I–T* properties of the initial ZnO tetrapods under DARK and UV illumination. Thus, the photoelectric response shows an increase, and the response and recovery times are noticeably slower compared to those of the aero-titania sensors. All photoelectric values for the tested aero-TiO_2_ sensors are presented in Table 2.

**Figure 6:**
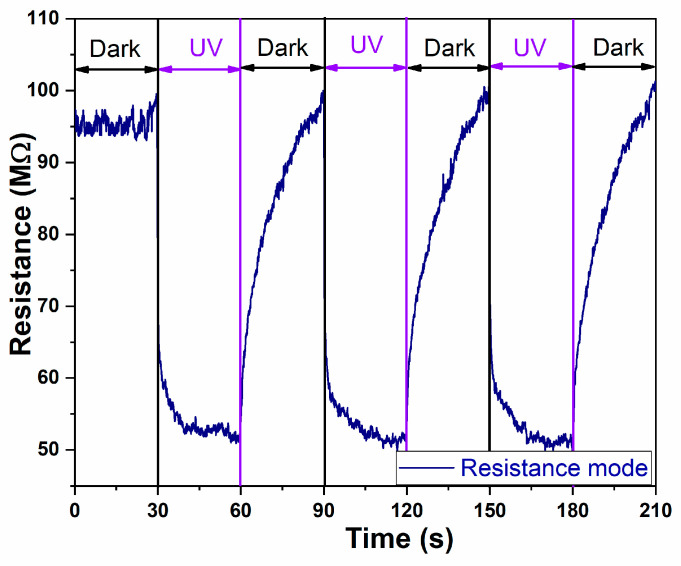
The UV time-dependent resistance changes in aero-titania for A2-aero-TiO_2_ device.

In Figure 6, the resistance response of the sensor under DARK and UV light illumination for the A2-aero-TiO_2_(HIM) device is presented. The resistance response under UV and DARK conditions demonstrates the typical behavior of an n-type semiconductor sensor [34]. Under UV illumination, a rapid decrease in electrical resistance is observed due to the oxygen desorption processes, which lead to increases in free carrier concentrations and an enhanced conductibility. After turning OFF the UV light, the resistance gradually recovers due to the oxygen molecule absorption process [35].

The stability of the UV sensor was evaluated over a period of seven days for the two-deposited-layer device (A2-aero-TiO_2_), and the results are presented in Figure S2 in the Supplementary Materials. The DARK current–time characteristics (increased from 30 s to 3 min) reveal that the sensor maintains nearly constant behavior throughout the testing period. From the day-by-day variation, it can be seen that both the DARK and UV illumination currents show slight fluctuations. Interestingly, the DARK current exhibits a moderate increase during Days 2 and 3, followed by a gradual decrease after Day 4, indicating minor changes in the film conductivity over time. We believe that this modification is mainly due to environmental factors such as humidity and temperature variations, as the measurements were performed in room temperature conditions. Nevertheless, these variations are relatively small, and the results confirm that the aero-titania sensor presented good stability properties.

Statistical testing using ultra-light materials with a high degree of porosity, such as aero-titania, is challenging. To address this limitation and minimize potential errors, all layers were deposited on the same device using the same batch of aero-titania. Therefore, the reported performance data are indicative and not supported by statistical analysis.

**Figure 7:**
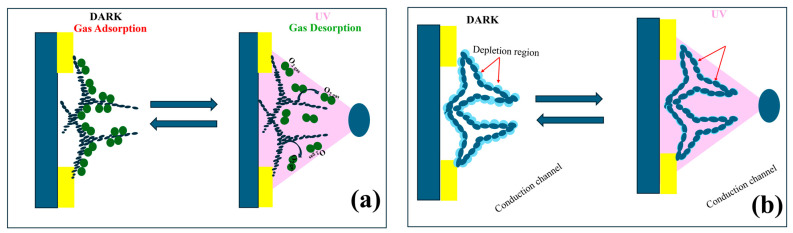
Schematic representation of space charge model in dark and under UV illumination of aero-titania; (**a**) Oxygen adsorption–desorption process; (**b**) surface depletion zone buildup under DARK and UV conditions.

The schematics of the sensing mechanism of aero-TiO_2_ devices under UV excitation are presented in Figure 7. They consists of two distinct schemes depicting the surface adsorption/desorption processes and the charge carrier dynamics at the aero-TiO_2_ heterojunction interface. The aero-titania UV sensor works as a chemiresistive device, exhibiting changes in electrical resistance due to the surface interactions with reactive chemical species under UV illumination [36]. Figure 7a presents the adsorption–desorption mechanism at the surface of nanoporous aero-TiO_2_. In the DARK condition, the oxygen molecules from the air are adsorbed onto the surface of the aero-titania material, trapping free electrons as shown in Equation (3) [30,37].

(3) $$O_{2\left(gas\right)}+e^{-}\to O_{2(ads)}^{-}$$

Under UV illumination, the photogenerated electron–hole pairs migrate to the surface of the material, where the holes mediate the desorption of O_2_-molecules (Equation (4)) [30].

(4) $$hv\to e^{-}+h^{+}$$

Equation (5) shows the mechanism of how the photogenerated holes help desorb the adsorbed oxygen species, releasing the trapped electrons back into the conduction band [30].

(5) $$O_{2(abs)}^{-}+h^{+}\to O_{2(desorp)}$$

Figure 7b illustrates the effect of adsorption–desorption dynamics on charge carriers. In DARK conditions, the oxygen adsorbed onto the aero-TiO_2_ surface leads to the formation of an electron depletion zone, which creates a potential barrier and results in low conductivity. Under UV illumination, desorption processes reduce the surface-adsorbed oxygen, leading to the reduction of the depletion layer and a significant increase in electrical conductivity. This dynamic change in conductivity directly influences the UV sensitivity of the device. Moreover, the response and recovery rates of the charge carriers determine the speed of the sensor’s operation. Additionally, the figure highlights the formation of conduction channels facilitated by the aero-material’s 3D structure [37,38,39].

## 3. Materials and Methods

### 3.1. Synthesis of Aero-TiO_2_ Materials

The aero-titania (aero-TiO_2_) material was obtained using the ALD technique to form ultrathin layers of TiO_2_ films on a sacrificial template consisting of an interconnected network of ZnO micro-tetrapods, as previously described elsewhere [18]. The TiO_2_ films with a thickness of 50 nm were deposited using a thermal ALD reactor equipped with a disk-like chamber (diameter = 300 mm, height = 7 mm). Titanium tetrachloride (TiCl_4_, Sigma-Aldrich, St. Louis, MO, USA) and H_2_O were used as the Ti precursor and as the oxygen source, respectively. During the deposition process, the substrate temperature was kept at 150 °C, and 20 sccm of high-purity N_2_ was used as the carrier gas during the reaction process. The optimized pulse and purge times were 0.2/120/0.015/120 s for one ALD cycle of TiO_2_ (TiCl_4_/N_2_/H_2_O/N_2_). After the deposition process, the removal of the sacrificial template of ZnO micro-tetrapods was realized at 800 °C in a flow of HCl gas and H_2_ in a horizontal reactor of a HVPE system. At a high temperature in the corrosive atmosphere, the ZnO core was decomposed, leaving only the ultrathin layer of TiO_2_ that retains the shape of the initial micro-tetrapodal interpenetrated network (Figure 1).

### 3.2. Fabrication of Aero-TiO_2_ Sensor for UV Detection

The sensor fabrication process involved dispersing a certain quantity of the aero-TiO_2_ in 50 µL of absolute ethanol. The interdigitated ceramic electrodes (10 × 10/10 × 20 mm, Shenzhen Zhifeng Electronics Co., Ltd., Shenzhen, China) were cleaned through ultrasonication for 20 min in an ethanol solution and then washed in distilled water, followed by drying under a vacuum. Before the deposition process, the electrodes were treated for 15 min by a UV Ozone Cleaner (Ossila Producer, Sheffield, UK) to remove all possible residues. The measuring platform has an active area of 0.7 cm^2^ and a width of 100 μm between the two gold contacts on the Au/Cr alumina substrate. A volume of 10 µL of the prepared suspension of aero-tetrapods in alcohol was deposited onto the measuring platform using a simple and facile spin-coating (WS-400-6NPPB Spin Coater-Laurell Technology Corporation, Lansdale, PA, USA) technique, conducted at 1000 rot for 10 s, and then dried on a hotplate at 100 °C for 10 min. In order to achieve a sufficient number of connections between aero-tetrapods that results in better electrical contact, the spin-coating process was repeated several times. Depending on the number of layers deposited, the samples were coded as A1-aero-TiO_2_ (one layer deposited), A2-aero-TiO_2_ (two layers deposited), and A3-aero-TiO_2_ (three layers deposited).

### 3.3. Characterization Techniques

#### 3.3.1. Morpho-Structural and Electrical Characterization

The morphology of the aero-materials was analyzed using scanning electron microscopy (SEM, TESCAN model, VEGA LMU, Brno–Kohoutovice, Czech Republic). This system is equipped with a tungsten filament electron source and integrated detectors for both secondary and backscattered electrons. The working conditions included an acceleration voltage of 15 kV, a beam current of approximately 32 pA, and working distances ranging from 14.21 to 18.99 mm. The crystallinity was assessed through the X-ray diffraction technique (XRD, PANalytical X’Pert PRO MPD Diffractometer, Almelo, The Netherlands) with Cu-K radiation in the range 2theta = 20–80°. UV-visible spectroscopy (UV-VIS, PerkinElmer Lambda 950 UV-Vis spectrophotometer, Shelton, CT, USA) coupled with an integrating sphere in the range 400–800 nm was employed. The electrical measurements of the aero-TiO_2_ material were performed using the Keithley 2450 SourceMeter SMU Instruments (Keithley Company, Cleveland, OH, USA).

#### 3.3.2. UV Analysis

The UV-sensing measurements were performed at ambient temperature in the dark and under UV irradiation at λ = 395 nm, using a commercial LED source (Alonfire Company, Model SV96, Guangdong, China). The distance between the UV source and measuring platform was about 10 cm, consistent with an intensity of 3500 µW cm^−2^ for high-intensity mode and 2000 µW cm^−2^ for low-intensity mode, respectively. The humidity of the ambient atmosphere during the measurements was approximately 35–40%.

## 4. Conclusions

A room temperature photodetector based on aero-titania deposited on interdigitated electrodes was developed and characterized. The aero-materials were obtained through the ALD of TiO_2_ ultrathin layers onto a ZnO sacrificial network consisting of interpenetrated micro-tetrapods. SEM images from after the material was exposed to high temperature treatment in an H_2_ flow exhibit the formation of the nanoporous hollow micro-tetrapods of aero-TiO_2_, with some traces of unetched ZnO rods. Imaging for the sequential depositions of the material onto the interdigitated electrodes using the spin-coating method clearly evidenced the need for an optimization of the deposition process in order to obtain a homogeneous tetrapod density. The XRD analysis confirms the formation of rutile-phase TiO_2_, and about 15% of wurtzite ZnO was incompletely etched in the H_2_ flow. Based on UV-vis spectroscopy, a slight extension of the absorption in the near-visible UV region suggests the possible formation of a core–shell ZnO-TiO_2_ structure that generated a modification of the optical response.

The sensitivity of the aero-titania sensor was demonstrated through dynamic current and resistance responses to the alternation of UV illumination. We also optimized the number of spin-coating layers to achieve the maximum UV photodetection performance, revealing that sensitivity is dependent on both the number of deposition cycles and the UV light intensity. Specifically, the highest sensitivity under high-intensity UV was approximately 23.6 for the A2-aero-TiO_2_ sensor, while, under low-intensity UV, the peak sensitivity was around 12.5 for the A1-aero-TiO_2_ sensor. The responsivity value was also calculated, showing that it is directly influenced by the optical power. The highest responsivity was obtained for the A1-aero-TiO_2_ sensor under low-intensity illumination, reaching about 1.23 × 10^−4^ A W^−1^ cm^−2^. The aero-titania sensor stability was evaluated for a seven-day period of time, indicating a consistent performance with only minor variations in dark and illumination currents. These slight changes are attributed to environmental influences, confirming that the sensor maintains a good electrical stability and reliable operation over time. Our findings highlight the importance of customizing the number of deposition layers for optimal sensitivity and responsivity based on light conditions.

## Figures and Tables

**Table 1:** Crystallite sizes determined using the Scherrer method and Williamson–Hall method, including anisotropy index and micro-strain calculations.

Compound	D_(hkl)_	Debye–Scherrer Method D (nm)	Debye–Scherrer Method Average D (nm)	Anisotropy Index (%)	Williamson–Hall Method Average D (nm)	Microstrain (ε) ×10^−3^
TiO_2_	D_(110)_	66	79	14.38	67	0.183124
	D_(101)_	94				
	D_(211)_	77				
ZnO	D_(110)_	240	262	23.73	473	−0.216963
	D_(002)_	347				
	D_(112)_	200				

**Table 2:** UV photodetector performances of aero-TiO_2_ sensors.

Sensor	Sensibility	Responsivity A W^−1^	Response Time (s)	Recovery Time (s)
A1-aero-TiO_2_ (HIM)	17.00	1.02 × 10^−4^	2.23	7.80
A1-aero-TiO_2_ (LIM)	12.50	1.23 × 10^−4^	2.31	6.90
A2-aero-TiO_2_ (HIM)	23.60	1.05 × 10^−4^	2.50	4.40
A2-aero-TiO_2_ (LIM)	9.63	1.04 × 10^−4^	2.70	3.20
A3-aero-TiO_2_ (HIM)	14.10	7.64 × 10^−5^	2.30	9.10
A3-aero-TiO_2_ (LIM)	8.87	9.79 × 10^−5^	2.10	6.10

## Data Availability

The original contributions presented in this study are included in the article/Supplementary Materials. Further inquiries can be directed to the corresponding authors.

## Supplementary Materials

The following supporting information can be downloaded at https://www.mdpi.com/article/10.3390/ijms262211035/s1.

## References

[1] Miller D.R., Akbar S.A., Morris P.A. (2014). Nanoscale metal oxide-based heterojunctions for gas sensing: A review. Sens. Actuators B Chem..

[2] Orha C., Nicolaescu M., Morariu M.-I., Galatonova T., Busuioc S., Lazau C., Bandas C. (2025). A Comprehensive Review on Aero-Materials: Present and Future Perspectives. Coatings.

[3] Ursaki V., Braniste T., Marangoci N., Tiginyanu I. (2025). Emerging aero-semiconductor 3D micro-nano-architectures: Technology, characterization and prospects for applications. Appl. Surf. Sci. Adv..

[4] Yang D., Gopal R.A., Lkhagvaa T., Choi D. (2021). Oxidizing agent impacting on growth of ZnO tetrapod nanostructures and its characterization. Environ. Res..

[5] Paisrisarn P., Yasui T., Zhu Z., Klamchuen A., Kasamechonchung P., Wutikhun T., Yordsri V., Baba Y. (2022). Tailoring ZnO nanowire crystallinity and morphology for label-free capturing of extracellular vesicles. Nanoscale.

[6] Li F., Meng Y., Kang X., Yip S., Bu X., Zhang H., Ho J.C. (2020). High-mobility In and Ga co-doped ZnO nanowires for high-performance transistors and ultraviolet photodetectors. Nanoscale.

[7] Abubakar S., Ying Chyi J.L., Tan S.T., Sagadevan S., Talib Z.A., Paiman S. (2021). Nanoscale domain imaging and the electromechanical response of zinc oxide nanorod arrays synthesized on different substrates. J. Mater. Res. Technol..

[8] Bakry M., Ismail W., Abdelfatah M., El-Shaer A. (2024). Low-cost fabrication methods of ZnO nanorods and their physical and photoelectrochemical properties for optoelectronic applications. Sci. Rep..

[9] Yi C., Yu Z., Ren Q., Liu X., Wang Y., Sun X., Yin S., Pan J., Huang X. (2020). Nanoscale ZnO-based photosensitizers for photodynamic therapy. Photodiagnosis Photodyn. Ther..

[10] Wang Y., Coppel Y., Lepetit C., Marty J.-D., Mingotaud C., Kahn M.L. (2021). Anisotropic growth of ZnO nanoparticles driven by the structure of amine surfactants: The role of surface dynamics in nanocrystal growth. Nanoscale Adv..

[11] Poschmann M.P.M., Siebert L., Lupan C., Lupan O., Schütt F., Adelung R., Stock N. (2023). Surface Conversion of ZnO Tetrapods Produces Pinhole-Free ZIF-8 Layers for Selective and Sensitive H_2_ Sensing Even in Pure Methane. ACS Appl. Mater. Interfaces.

[12] Padmanaban D.b., Maguire P., Mariotti D. (2024). Non-equilibrium defect chemistry in oxygen-rich zinc oxide nano-tetrapods synthesized using atmospheric pressure microplasma. J. Mater. Chem. A.

[13] Dmytruk A., Dmitruk I., Shynkarenko Y., Belosludov R., Kasuya A. (2017). ZnO nested shell magic clusters as tetrapod nuclei. RSC Adv..

[14] Yan L., Uddin A., Wang H. (2015). ZnO Tetrapods: Synthesis and Applications in Solar Cells. Nanomater. Nanotechnol..

[15] Veys E., Makower L., Williamson M., Saure L.M., Adelung R., Schütt F., Pugno N.M., Marrow T.J. (2023). In situ observation of compressive deformation of an interconnected network of zinc oxide tetrapods. Scr. Mater..

[16] Albulescu D., Ursu D., Rusnac L.-M., Nitu S., Miclau M., Vajda M. (2022). Investigation of UV Dye-Sensitized Solar Cells Based on Water Electrolyte: A New Insight for Wavelength-Selective Greenhouse. Crystals.

[17] Chandoliya R., Sharma S., Sharma V., Joshi R., Sivanesan I. (2024). Titanium Dioxide Nanoparticle: A Comprehensive Review on Synthesis, Applications and Toxicity. Plants.

[18] Ciobanu V., Ursaki V.V., Lehmann S., Braniste T., Raevschi S., Zalamai V.V., Monaico E.V., Colpo P., Nielsch K., Tiginyanu I.M. (2022). Aero-TiO2 Prepared on the Basis of Networks of ZnO Tetrapods. Crystals.

[19] Wyckoff R.W.G. (1963). Crystal Structures.

[20] Baur W.H., Khan A.A. (1971). Rutile-type compounds. IV. SiO_2_, GeO_2_ and a comparison with other rutile-type structures. Acta Crystallogr. Sect. B.

[21] Holzwarth U., Gibson N. (2011). The Scherrer equation versus the 'Debye-Scherrer equation'. Nat. Nanotechnol..

[22] Srikant V., Clarke D.R. (1998). On the optical band gap of zinc oxide. J. Appl. Phys..

[23] Li D., Jiang X., Zhang Y., Zhang B., Pan C. (2013). A novel route to ZnO/TiO_2_ heterojunction composite fibers. J. Mater. Res..

[24] Mathew S., Ganguly P., Rhatigan S., Kumaravel V., Byrne C., Hinder S.J., Bartlett J., Nolan M., Pillai S.C. (2018). Cu-Doped TiO_2_: Visible Light Assisted Photocatalytic Antimicrobial Activity. Appl. Sci..

[25] Lazau C., Nicolaescu M., Orha C., Şerban V., Bandas C. (2022). Self-Powered Photodetector Based on FTO/n-TiO_2_/p-CuMnO_2_ Transparent Thin Films. Materials.

[26] Ranjith K.S., Rajendra Kumar R.T. (2016). Facile construction of vertically aligned ZnO nanorod/PEDOT:PSS hybrid heterojunction-based ultraviolet light sensors: Efficient performance and mechanism. Nanotechnology.

[27] Gröttrup J., Postica V., Smazna D., Hoppe M., Kaidas V., Mishra Y.K., Lupan O., Adelung R. (2017). UV detection properties of hybrid ZnO tetrapod 3-D networks. Vacuum.

[28] Postica V., Paulowicz I., Lupan O., Schütt F., Wolff N., Cojocaru A., Mishra Y.K., Kienle L., Adelung R. (2019). The effect of morphology and functionalization on UV detection properties of ZnO networked tetrapods and single nanowires. Vacuum.

[29] Lin H., Wei L., Wu C., Chen Y., Yan S., Mei L., Jiao J. (2016). High-Performance Self-powered Photodetectors Based on ZnO/ZnS Core-Shell Nanorod Arrays. Nanoscale Res. Lett..

[30] Nagpal R., Lupan C., Bîrnaz A., Sereacov A., Greve E., Gronenberg M., Siebert L., Adelung R., Lupan O. (2024). Multifunctional Three-in-One Sensor on t-ZnO for Ultraviolet and VOC Sensing for Bioengineering Applications. Biosensors.

[31] Ilickas M., Mardosaite R., Cesano F., Cravanzola S., Barolo C., Scarano D., Viscardi G., Rackauskas S. (2024). ZnO tetrapod morphology influence on UV sensing properties. Nanotechnology.

[32] Elsaeedy H.I., Qasem A., Yakout H.A., Mahmoud M. (2021). The pivotal role of TiO_2_ layer thickness in optimizing the performance of TiO_2_/P-Si solar cell. J. Alloys Compd..

[33] Chabri S., Dhara A., Show B., Adak D., Sinha A., Mukherjee N. (2016). Mesoporous CuO–ZnO p–n heterojunction based nanocomposites with high specific surface area for enhanced photocatalysis and electrochemical sensing. Catal. Sci. Technol..

[34] Zhao L., Sun S., Zhang S., Li X. (2024). UV activated formaldehyde gas sensing based on gold decorated ZnO@ In2O3 hollow nanospheres at room temperature. Sens. Actuators B Chem..

[35] Thepnurat M., Chairuangsri T., Hongsith N., Ruankham P., Choopun S. (2015). Realization of Interlinked ZnO Tetrapod Networks for UV Sensor and Room-Temperature Gas Sensor. ACS Appl. Mater. Interfaces.

[36] Nunes D., Fortunato E., Martins R. (2022). Flexible nanostructured TiO_2_-based gas and UV sensors: A review. Discov. Mater..

[37] Shree S., Postica V., Voß L., Lupan C., Mishra Y.K., Kienle L., Adelung R., Lupan O. (2025). Optimization of T-ZnO Process for Gas and UV Sensors. ACS Appl. Electron. Mater..

[38] Knoepfel A., Liu N., Hou Y., Sujani S., dos Reis B.R., White R., Wang K., Poudel B., Gupta S., Priya S. (2022). Development of Tetrapod Zinc Oxide-Based UV Sensor for Precision Livestock Farming and Productivity. Biosensors.

[39] Sahu S., Bhattacharjee M. (2025). Nanostructured ZnO thin film-based flexible printed sensor for high-performance UV detection. Sens. Actuators A Phys..

