# Supplementary material for: Room Temperature UV Photodetector Based on Aero-Titania
Source: Int J Mol Sci. 2025 Nov 14;26(22):11035. doi: 10.3390/ijms262211035 (PMC12652746; doi:10.3390/ijms262211035)
Supplement: Supplementary file 1 [file ijms-26-11035-s001.zip › ijms-3909582-supplementary.pdf]

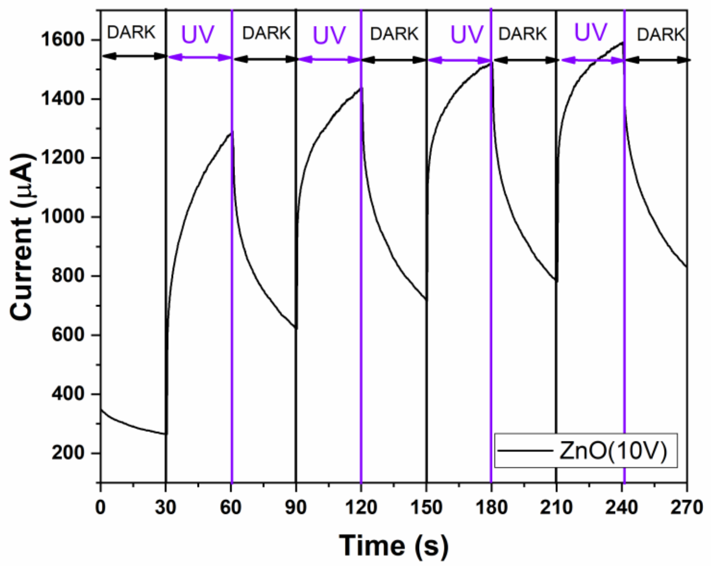

Figure S1. The UV time-dependent current response of ZnO tetrapods

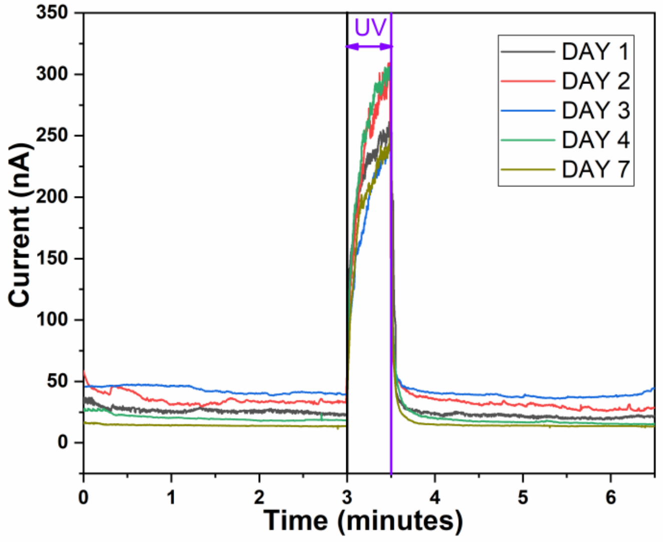

Figure S2. The stability of the A2-aero-TiO_2 device evaluated for seven days in room temperature conditions
